# MYBPC3 deficiency in cardiac fibroblasts drives their activation and contributes to fibrosis

**DOI:** 10.1038/s41419-022-05403-6

**Published:** 2022-11-10

**Authors:** Xiaodong Zou, Hongsheng Ouyang, Feng Lin, Huanyu Zhang, Yang Yang, Daxin Pang, Renzhi Han, Xiaochun Tang

**Affiliations:** 1 Jilin Provincial Key Laboratory of Animal Embryo Engineering, College of Animal Sciences, Jilin University, Changchun, Jilin Province People’s Republic of China; 2 Chongqing Research Institute of Jilin University, Chongqing, China; 3 Department of Surgery, Davis Heart and Lung Research Institute, Biomedical Sciences Graduate Program, Biophysics Graduate Program, The Ohio State University Wexner Medical Center, Columbus, OH 43210 USA

**Keywords:** Cardiac hypertrophy, Mechanisms of disease

## Abstract

Genetic mutations in the *MYBPC3* gene encoding cardiac myosin binding protein C (cMyBP-C) are the most common cause of hypertrophic cardiomyopathy (HCM). Myocardial fibrosis (MF) plays a critical role in the development of HCM. However, the mechanism for mutant *MYBPC3*-induced MF is not well defined. In this study, we developed a R495Q mutant pig model using cytosine base editing and observed an early-onset MF in these mutant pigs shortly after birth. Unexpectedly, we found that the “cardiac-specific” *MYBPC3* gene was actually expressed in cardiac fibroblasts from different species as well as NIH3T3 fibroblasts at the transcription and protein levels. CRISPR-mediated disruption of *Mybpc3* in NIH3T3 fibroblasts activated nuclear factor κB (NF-κB) signaling pathway, which increased the expression of transforming growth factor beta (TGF-β1) and other pro-inflammatory genes. The upregulation of TGF-β1 promoted the expression of hypoxia-inducible factor-1 subunit α (HIF-1α) and its downstream targets involved in glycolysis such as GLUT1, PFK, and LDHA. Consequently, the enhanced aerobic glycolysis with higher rate of ATP biosynthesis accelerated the activation of cardiac fibroblasts, contributing to the development of HCM. This work reveals an intrinsic role of MYBPC3 in maintaining cardiac fibroblast homeostasis and disruption of MYBPC3 in these cells contributes to the disease pathogenesis of HCM.

## Introduction

Hypertrophic cardiomyopathy (HCM) is a heterogeneous group of diseases affecting people of both genders and of various ethnic and racial origins [1]. HCM is mainly caused by autosomal dominant mutations in sarcomere genes and disseminated with a prevalence of about 1:500–1:200 in the general population [2]. The clinical manifestations of HCM include left ventricular (LV) hypertrophy, myocardial hypercontractility, reduced compliance, myofibrillar disarray, and fibrosis [3]. During the last two decades, a wealthy body of evidence revealed *MYBPC3* as the most frequently mutated HCM gene, representing about 40–50% of all HCM mutations [4]. The *MYBPC3* gene encodes cardiac myosin binding protein C (cMyBP-C), a flexible, rod-like protein that is a key component of the cardiac sarcomere. Among HCM patients with genetic defects in *MYBPC3*, 90% of mutations are heterozygous frameshift, nonsense, or splice site mutations that result in premature termination codons (PTCs) and truncated cMyBP-C protein [5]. Previous studies reported that the knockout of *mybpc3* caused zebrafish displayed significant morphological heart alterations at systolic and diastolic states at the larval stages, and revealed that an impaired actin cytoskeleton organization as the main dysregulating factor associated with the early ventricular cardiac hypertrophy in the zebrafish *mypbc3* HCM model [6]. Moreover, the loss of cMyBP-C protein results in left ventricular dilation, cardiac hypertrophy and impaired ventricular function in *Mybpc3* null mice [7]. Microarray analysis on left ventricles of wild-type (WT) and cMyBP-C^−/−^ mice at postnatal day (PND) 1 and 9 (before and after the appearance of an overt HCM phenotype) identified the importance of extracellular matrix pathways in hypertrophic growth and early dysregulation of potassium channels [8].

Cardiac fibrosis is a hallmark of most myocardial pathologies with limited treatment options [9]. Cardiac fibrosis-related diseases are associated with a high mortality rate and the morbidity increases with age [10]. Myocardial fibrosis (MF), as evidenced by the proliferation of cardiac fibroblasts (CFs) and excessive deposition of collagen in myocardial tissue [11], has proven to be an important marker and determinant in the pathogenesis of HCM [12].

Several signaling pathways have been implicated in the early activation of CFs. The TGF-β family of growth factors are the most extensively studied mediators of fibroblast activation, of which TGF-β1 plays a crucial role in pathological fibrosis [13]. The canonical pathway of TGF-β1 signaling involves the phosphorylation of Smad2/3, which then binds Smad4, translocates into the nucleus, and acts as a transcription factor, inducing the activation of numerous profibrotic genes [14]. In addition to the Smad-mediated pathways, TGF-β1 can also induce noncanonical signaling that involves the activation of TGF-β-activated kinase (TAK) 1, which is thought to contribute to pathological cardiac remodeling. Cardiac overexpression of constitutively active TAK1 induces cardiac hypertrophy and heart failure [15]. A growing body of evidence suggests that the noncanonical pathway may actually be the predominant driving force [16]. The TGF-β noncanonical signaling pathway is thought to propagate primarily through the type II TGF-β receptor, as supported by the evidence that cardiomyocyte-specific deletion of the type II TGF-β receptor resulted in reduced fibrosis and remodeling in the transverse aortic constriction model of heart failure [17].

In this study, we generated a pig model of HCM with R495Q mutation in *MYBPC3* engineered with cytosine base editor (CBE) and observed severe MF in these mutant piglets soon after birth. Unexpectedly, we found that *MYBPC3* and several other related myocardial genes were expressed in fibroblasts. We further showed that CRISPR-mediated genetic disruption of *MYBPC3* in fibroblasts promoted their activation into myofibroblasts via the NF-κB/TGF-β1/HIF-1α aerobic glycolysis signal cascade.

## Results

### *MYBPC3* R495Q mutation pigs developed premature myocardial fibrosis

**Fig. 1:**
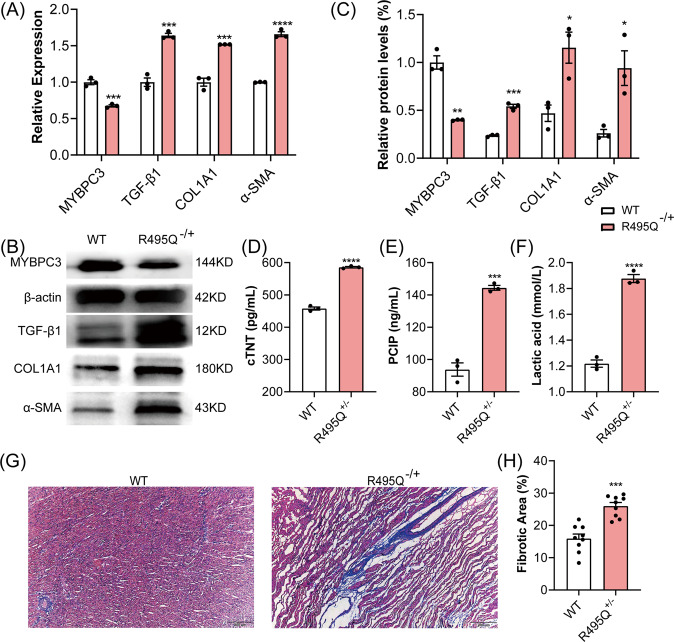
MYBPC3-R495Q^+/−^ pigs developed severe myocardial fibrosis, concomitant with reduced *MYBPC3* gene expression and elevated lactic acid concentrations. **A**–**C** The transcript (**A**) and protein (**B**, **C**) expression levels of MYBPC3, TGF-β1, COL1A1, and α-SMA in MYBPC3-R495Q^+/−^ pigs and the age-matched wild-type pigs. ***P < 0.001. **D**–**F** Measurements of serum cTNT (**D**), PCIP (**E**), and lactic acid (**F**) in WT and MYBPC3-R495Q^+/−^ pigs. ***P < 0.001; ****P < 0.0001. **G**, **H** Masson trichrome staining and quantification of cardiac fibrosis in WT and MYBPC3-R495Q^+/−^ pigs. ***P < 0.001. *N* = 3 per genotype.

First, we established *MYBPC3* R495Q mutant porcine fetal fibroblasts (PFFs) by CBE, cell monoclonal techniques and Sanger sequencing (Supplemental Fig. S1a). Electroporation of CBE and the MYBPC3-targeting sgRNA resulted in the conversion of CCG to CTG in the exon 16 of pig *MYBPC3* (Supplemental Fig. S1b). The R495Q heterozygous PFFs were used to generate the mutant pigs via somatic cell nuclear transfer (SCNT). In total, three pregnant surrogates were carried to term, and six piglets were delivered. All piglets were genotyped by sequencing (Supplemental Fig. S1c), and three of newborns carried mutations at the target locus. Sequencing analysis of the six top predicted off-target sites showed no detectable editing (Supplemental Fig. S1d). The *MYBPC3* R495Q mutation piglets began to die soon after birth, with all three dead within 10 days, whereas all wild-type piglets survived. Quantitative RT-PCR (qRT-PCR) analysis showed that the expression of *MYBPC3* was significantly decreased in cardiac muscle of *R495Q* mutant pigs as compared with WT littermates (Fig. 1A). Consistently, Western blot analysis showed that the cMyBP-C protein was significantly reduced in the R495Q mutant pig hearts (Fig. 1B, C and Supplemental Fig. S7). Serological examination showed that the high sensitivity cardiac troponin T (cTNT) and procollagen type I carboxy-terminal propeptide (PICP), which are highly sensitive markers for myocardial injury and fibrosis, respectively [18, 19], were significantly elevated in the mutant pigs (Fig. 1D, E). In addition, the transcript and protein expression levels of the fibrosis-related genes such as type I collagens α1 (COL1A1), α-Smooth muscle actin (α-SMA) and profibrotic cytokines (TGF-β1) were significantly up-regulated in the mutant pig hearts (Fig. 1A–C). We also observed inflammatory infiltrates in heart tissue sections (Supplemental Fig. S1e) and the abnormally elevated pro-inflammatory cytokines in serum samples of the mutant pigs (Supplemental Fig. S1f–i). Remarkably, lactic acid concentrations in both serum (Fig. 1F) and myocardial muscles (Supplemental Fig. S1j) of the mutant pigs were highly elevated. Furthermore, Masson’s trichrome staining of paraffin sections showed severe myocardial fibrosis in the mutant pigs (Fig. 1G, H). Taken together, these results demonstrated that the *MYBPC3* R495Q mutation led to the development of severe myocardial fibrosis and inflammation.

### MYBPC3 is expressed in cardiac fibroblasts

**Fig. 2:**
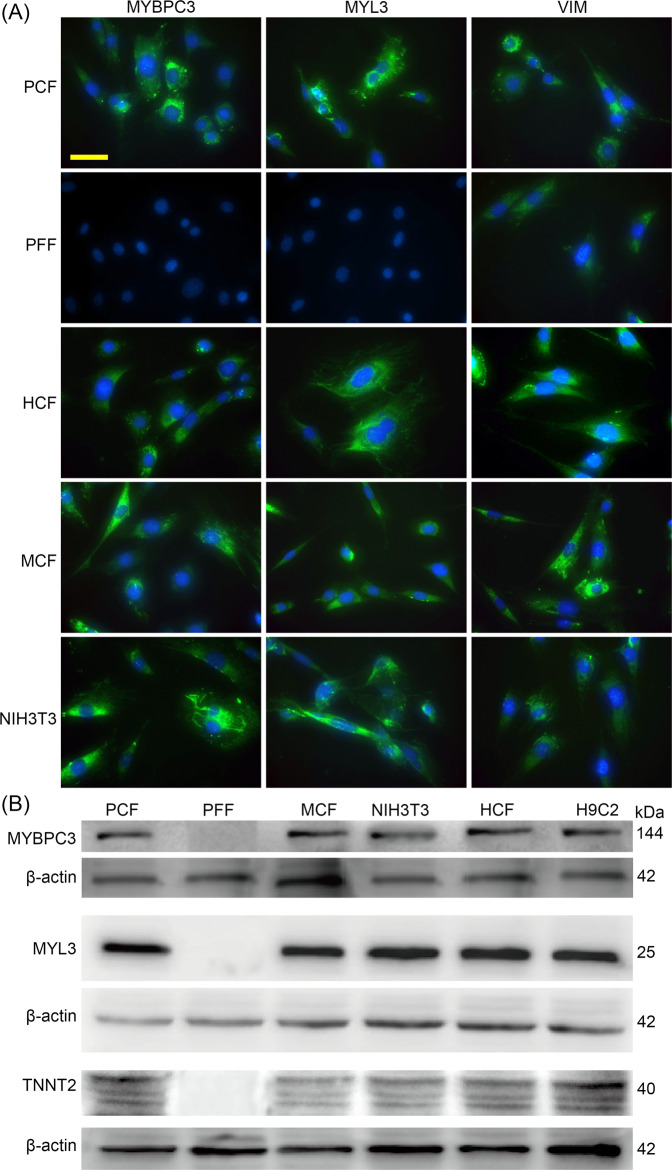
*MYBPC3* and related myocardial proteins are expressed in cardiac fibroblasts of various species and NIH3T3 cells. **A** Immunofluorescence staining of MYBPC3, MYL3, and VIM in primary cardiac fibroblasts from pig (PCF), human (HCF), and mouse (MCF), as well as NIH3T3 fibroblasts. MYBPC3 and MYL3 were found to be negative in porcine fetal fibroblasts (PFF). **B** Western blot analysis of MYBPC3, MYL3, TNNT2 in various cardiac fibroblasts, PFF and NIH3T3 cells. H9C2 lysate was used as a positive control.

The early onset MF phenotype in the mutant pigs suggests a potential intrinsic role of *MYBPC3* deficiency in regulating fibroblast trans-differentiation. We first examined the expression of MYBPC3 in the major cell types within mouse heart, including mouse cardiac myocytes (CM), fibroblasts (CF), microvascular endothelial cells (MVET), aortic smooth muscle cells (ASM) and coronary artery smooth muscle cells (CASM). The CM and CF were isolated from neonatal C57BL/6J mice (day 1–3), while MVET, ASM, and CASM were isolated from 3–6 weeks C57BL/6J mice. Quantitative RT-PCR showed that *Mybpc3* was expressed in CM and CF but not in other cell types (Supplemental Fig. S2A). Western blot analysis confirmed that the MYBPC3 protein was expressed in CF, at ~18% of that in CM (Supplemental Fig. S2B, C). MYBPC3 protein was not detectable in other cell types. We then examined the expression of *MYBPC3* in primary porcine cardiac fibroblasts (PCF). Immunofluorescence staining showed that both MYBPC3 and myosin light chain 3 (MYL3) were readily detectable in WT PCFs, which were also positive for the fibroblast marker vimentin (VIM) (Fig. 2A). The fluorescence signals were specific as we did not detect their expression in PFFs. To examine whether the expression of MYBPC3 and MYL3 in cardiac fibroblasts are species-specific, we analyzed their expression in primary human cardiac fibroblasts (HCF) and mouse cardiac fibroblasts (MCF) and found that both MYBPC3 and MYL3 were expressed in these cells (Fig. 2A). Moreover, we found that MYBPC3 and MYL3 were expressed in NIH3T3 cells, a commonly used mouse embryonic fibroblast cell line (Fig. 2A). Consistently, Western blot analysis confirmed the expression of the cardiac proteins MYBPC3, MYL3 and TNNT2 in NIH3T3 fibroblasts and primary mouse, pig and human cardiac fibroblasts, but not in PFFs (Fig. 2B and Supplemental Fig. S8).

### Loss of MYBPC3 promotes fibroblast differentiation

To understand the role of MYBPC3 in fibroblasts, we utilized the CRISPR/Cas9 system to knockout *Mybpc3* gene in NIH3T3 mouse fibroblasts (MYBPC3-KO). A guide RNA (gRNA) targeting the exon 2 of mouse *Mybpc3* gene was transfected into NIH3T3 cells together with the Cas9-expressing plasmid (Supplemental Fig. S3a). Sequencing of the genomic DNA PCR amplicon showed efficient generation of indels at the gRNA target site (Supplemental Fig. S3b) but not at the top 5 predicted off-target sites (Supplemental Fig. S3c). The *Mybpc3* transcript and protein expression was disrupted in MYBPC3-KO fibroblasts (Supplemental Fig. S3e, f and S15).

**Fig. 3:**
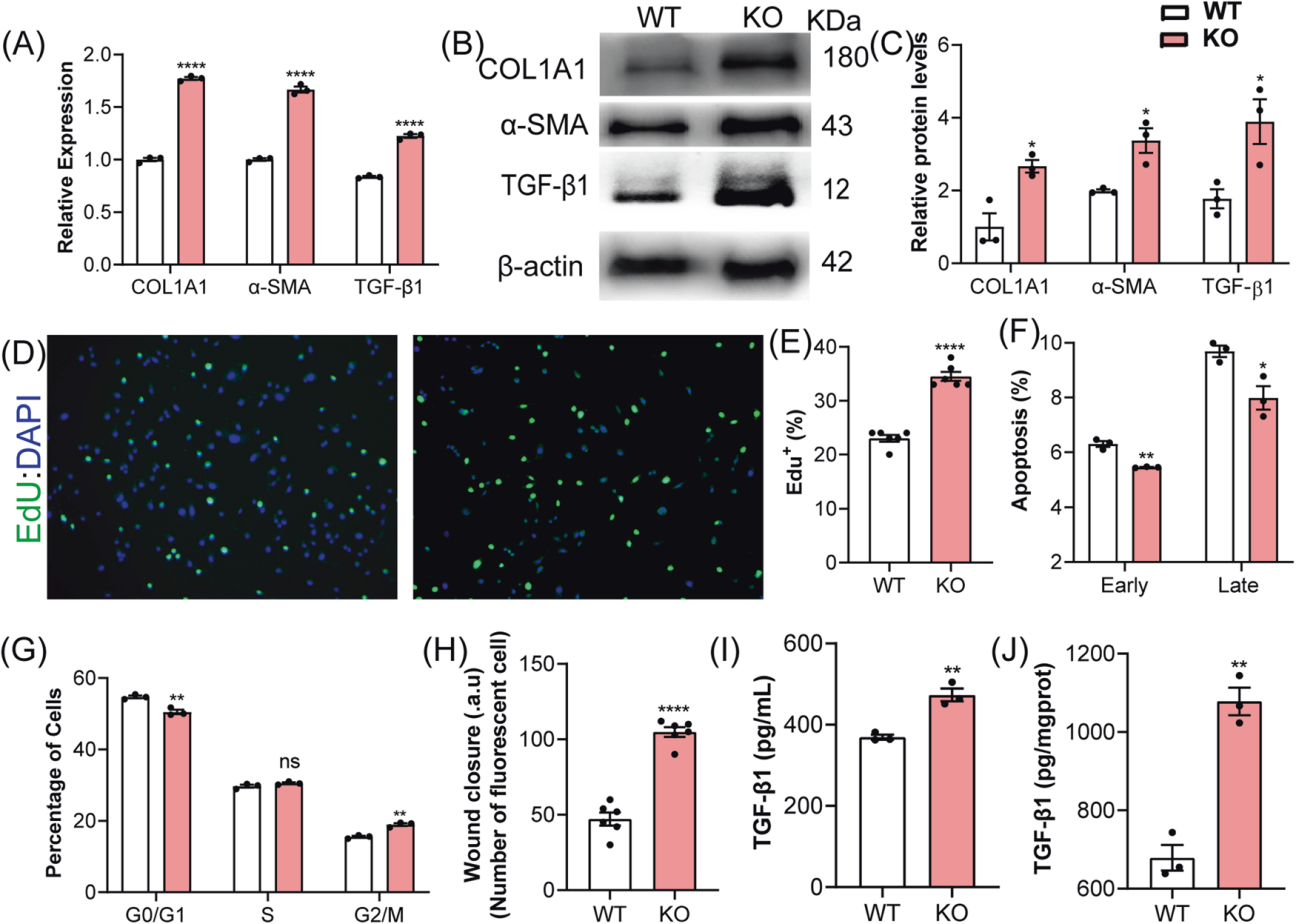
*Mybpc3* deficiency enhanced myofibroblasts conversion. **A**–**C** The transcript (**A**) and protein (**B**, **C**) expression of COL1A1, α-SMA, and TGF-β1 in WT and MYBPC3-KO fibroblasts. ****P < 0.0001. **D**–**F** The deficiency of *Mybpc3* gene increased fibroblast proliferation and reduced apoptosis. *P < 0.05; **P < 0.01; ***P < 0.001. **G** Cell cycle analysis showed that *Mybpc3* disruption increased cells in the G2/M phase while slightly reducing cells in the G0/G1 phase. ns, not significant; **P < 0.01. **H** Measurement of fibroblast migration by the cell wound scratch assay. ****P < 0.0001. **I**, **J** Measurement of TGF-β1 in the culture supernatant and cell lysate of WT and MYBPC3-KO fibroblasts. **P < 0.01.

Next, we examined whether *Mybpc3* disruption could induce fibroblast activation. As shown in Fig. 3A–C and Supplemental Fig. S9, *Mybpc3* disruption significantly increased the transcription and protein expression of COL1A1 and α-SMA, the markers of myofibroblasts. Moreover, *Mybpc3* disruption led to accelerated proliferation and reduced apoptosis, as evidenced by EdU staining and apoptosis analysis, respectively (Fig. 3D–F and Supplemental Fig. S4a). In addition, *Mybpc3* disruption increased cells in the G2/M phase while slightly reducing cells in the G0/G1 phase (Fig. 3G and Supplemental Fig. S4b). The wound scratch assay showed that *Mybpc3* deficiency enhanced the migration of NIH3T3 fibroblasts (Fig. 3H and Supplemental Fig. S4c). Finally, the secretion of TGF-β1 into the culture medium and its expression in cell lysate were significantly increased (Fig. 3I, J). Together, these data suggest that *Mybpc3* disruption induces fibroblast activation.

### Loss of MYBPC3 enhances aerobic glycolysis in fibroblasts

**Fig. 4:**
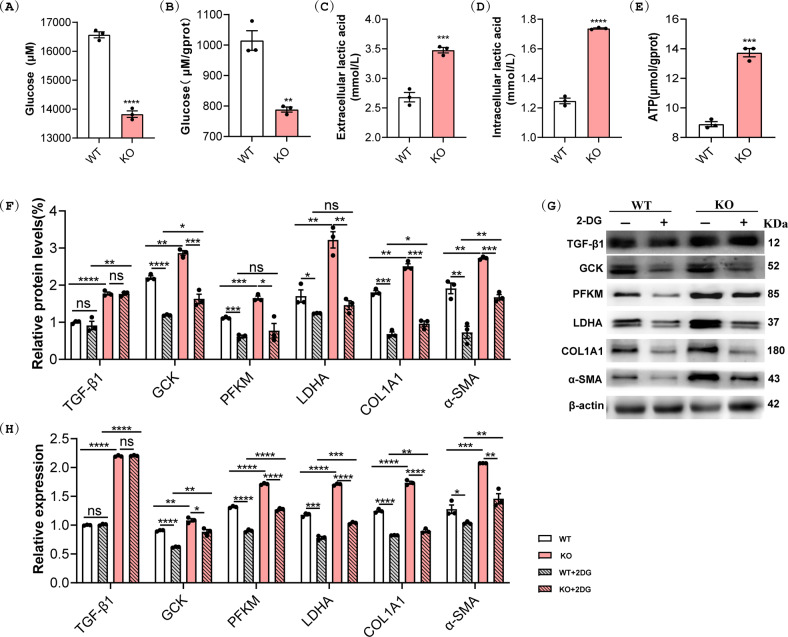
*Mybpc3* disruption enhanced aerobic glycolysis in fibroblasts. **A**–**E** Analysis of glycolytic flux by measurements of glucose concentrations (**A**, **B**), lactate secretion (**C**, **D**), and ATP production (**E**). **P < 0.01; ***P < 0.001; ****P < 0.0001. **F**, **G** Western blot analysis of key glycolytic enzymes in WT and MYBPC3-KO cells treated with or without 2-DG (5 mM). **H** qRT-PCR analysis of key glycolytic enzymes genes in WT and MYBPC3-KO cells treated with or without 2-DG (5 mM). *P < 0.05; **P < 0.01; ***P < 0.001; ****P < 0.0001; ns, not significant.

Previous research demonstrated that glycolytic reprogramming facilitate progression to, and maintenance of, the transformed myofibroblast state and that enhances contractility and cellular migration. Similarly, we observed that the glucose concentrations in the culture medium and cell lysate of MYBPC3-KO fibroblasts were decreased while lactic acid levels were increased more rapidly than the control fibroblasts (Fig. 4A–D). ATP production was also elevated in MYBPC3-KO fibroblasts (Fig. 4E). Moreover, the expression of GCK, PFKM and LDHA, the key enzymes involved in glycolysis, was significantly increased at both the transcriptional and translational levels in MYBPC3-KO fibroblasts as compared to WT controls (Fig. 4F–H and Supplemental Fig. S10). Treatment with the glucose analog 2-deoxy-d-glycose (2-DG) to inhibit glycolysis dramatically reduced the expression of glycolysis genes and fibrosis marker genes (COL1A1 and α-SMA) but had no impact on the expression of TGF-β1 (Fig. 4F–H). These results suggest that the loss of *Mybpc3* gene is a driving force for metabolic reprogramming in fibroblasts.

### Loss of MYBPC3 increases the expression of HIF-1α via TGF-β1 in fibroblasts

**Fig. 5:**
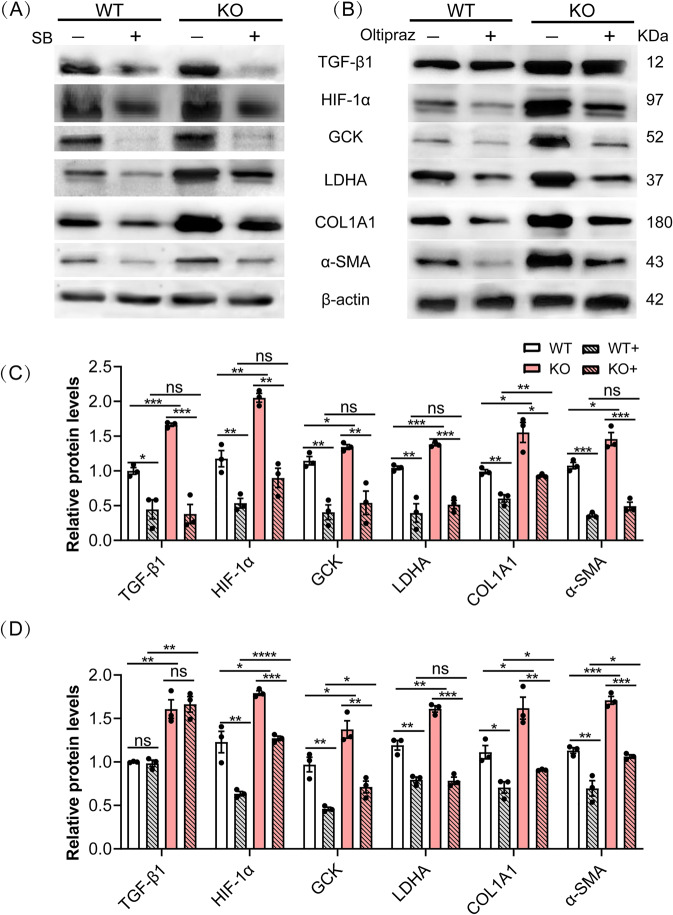
*Mybpc3* deficiency promoted HIF-1α expression in fibroblasts. **A**, **C** Western blot analysis of HIF-1α and the related signaling molecules in WT and MYBPC3-KO fibroblasts treated with or without SB-431542 (10 µM). **B**, **D** Western blot analysis of HIF-1α and the related signaling molecules in WT and MYBPC3-KO fibroblasts treated with or without Oltipraz (10 µM). *P < 0.05; **P < 0.01; ***P < 0.001; ****P < 0.0001; ns, not significant.

A potential crosstalk between HIF-1α and TGF-β1 has been proposed in driving fibrosis [20]. The expression of HIF-1α was significantly increased in MYBPC3-KO fibroblasts as compared with WT control (Fig. 5A–C). Treatment with SB-431542, a selective inhibitor of TGF-β type I receptor, dramatically suppressed the expression of HIF-1α in MYBPC3-KO fibroblasts (Fig. 5A, C and Supplemental Fig. S11), while the selective inhibitor of HIF-1α, Oltipraz, did not affect the expression of TGF-β1 (Fig. 5B, D and Supplemental Fig. S12). Both SB-431542 and Oltipraz attenuated glycolysis and fibrosis genes (Fig. 5A–D). These data suggest that the disruption of *Mybpc3* increased the expression of HIF-1α via TGF-β1 in fibroblasts.

### Loss of *Mybpc3* activates TGF-β1 via NF-κB signaling pathway

**Fig. 6:**
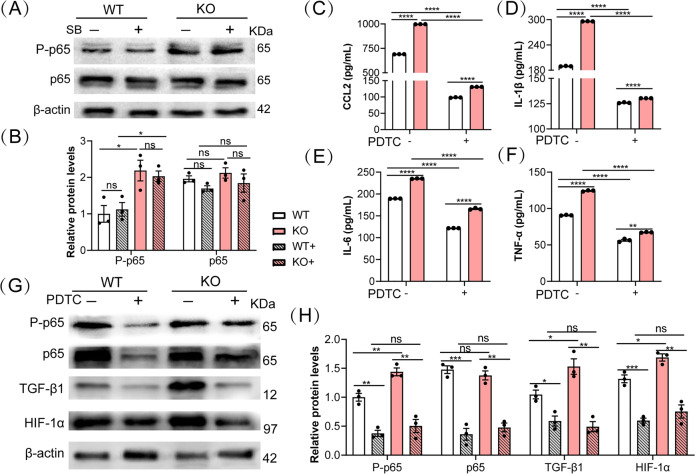
Genetic disruption of *Mybpc3* enhanced NF-κB signaling pathway in fibroblasts. **A**, **B** Western blotting analysis of phosphorylated and total p65 in WT and MYBPC3-KO fibroblasts treated with or without SB-431542 (10 µM). **C**–**F** ELISA measurements of the cytokine levels of CCL2, IL-1β, IL-6, and TNF-α in culture supernatant of WT and MYBPC3-KO fibroblasts treated with or without PDTC (10 µM). **G**, **H** Western blotting analysis of phosphorylated and total p65, TGF-β1, and HIF-1α in WT and MYBPC3-KO fibroblasts treated with or without PDTC (10 µM). *P < 0.05; **P < 0.01; ***P < 0.001; ****P < 0.0001; ns, not significant.

TGF-β1 and NF-κB were reported to be involved in liver fibrosis [21]. The cross-talk between TGF-β and NF-κB signaling pathways was mediated through TAK1 and SMAD7 in a subset of head and neck cancers [22]. First, we examined the NF-κB signaling pathway in WT and MYBPC3-KO fibroblasts. The phosphorylated p65, an indicator of NF-κB activity, was found to be increased in MYBPC3-KO fibroblasts and the TGF-β1 inhibitor SB-431542 did not impact the activation of NF-κB signaling pathway (Fig. 6A, B and Supplemental Fig. S13). NF-kB plays a critical role in controlling the expression of pro-inflammatory cytokines. We found that the levels of CCL2, IL-1β, IL-6, and TNF-α in both culture media and cell lysates of MYBPC3-KO fibroblasts were all significantly increased in MYBPC3-KO fibroblasts, which were significantly inhibited by the NF-κB signaling pathway inhibitor, PDTC (Fig. 6C–F and Supplemental Fig. 5). PDTC also significantly reduced the expression of TGF-β1 and HIF-1α (Fig. 6G, H and Supplemental S14), suggesting that they are the downstream signaling of NF-κB. Taken together, these results suggest that *Mybpc3* deficiency triggers the NF-κB-mediated signaling cascade in fibroblasts to drive their activation.

## Discussion

**Fig. 7:**
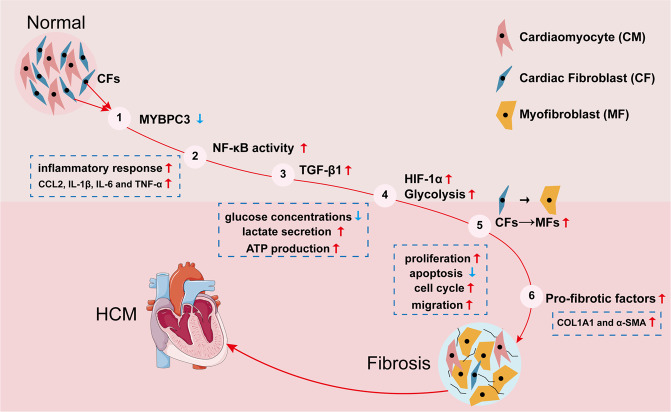
MYBPC3 deficiency up-regulated NF-κB/TGF-β1/HIF-1α/aerobic glycolysis signaling cascade in cardiac fibroblasts, contributing to HCM myocardial fibrosis. *MYBPC3* deficiency activated NF-κB signal pathway, which increased TGF-β1, HIF-1α, and aerobic glycolysis signal, leading to premature myocardial fibrosis in HCM.

In this study, we generated a pig model of HCM with *MYBPC3* mutation by CRISPR/Cas9 gene editing technology and demonstrated that the MYBPC3 mutant pigs developed premature MF. We unexpectedly found that MYBPC3 and other myofilament proteins were expressed in primary cardiac fibroblasts and NIH3T3 fibroblasts. We generated MYBPC3-KO NIH3T3 fibroblasts to dissect the signaling pathways involved in fibroblast activation. Our results revealed a novel fibroblast-intrinsic mechanism that links *MYBPC3* deficiency to the activation of NF-κB-mediated signaling cascade through TGF-β1, HIF-1α, and aerobic glycolysis, contributing to the development of premature MF in HCM (Fig. 7).

In this study, we demonstrated that *MYBPC3* gene mutations activated the NF-κB signal pathway. NF-κB is a key molecule in regulating TGF-β1 levels, and a potential crosstalk mechanism between NF-κB and TGF-β/Smad has been proposed in hepatic macrophages [23]. It has been reported that TGF-β1 and NF-κB were both involved in liver fibrosis [24]. The expression of TGF-β1 and the nuclear translocation of NF-κB were required in liver cirrhosis [25]. In renal fibrosis, the loss of *Chop* gene represses Hmgb1/TLR4 signal pathway, leading to repressed NF-κB transcriptional activity along with suppressed IL-1β production, and reduced TGF-β1 production and PI3K/Akt activity to attenuate the development of fibrosis [26].

Our studies also revealed that mutations in *MYBPC3* enhances HIF-1α expression and aerobic glycolysis. HIF-1α is a key transcription factor in response to chronic hypoxia and participates in fibrotic diseases, such as systemic sclerosis (SSc) [27]. Under hypoxic conditions, HIF-1α stably accumulates in the cytoplasm, and then transfers to the nucleus to form HIF-1α/ARNT dimer, and initiates the transcription of target genes, such as glycolytic genes (GLUT1, PFK, LDHA) [28]. However, hypoxia-independent mechanisms to regulate HIF-1α have also been proposed. In certain tumors, high levels of HIF-1α are observed in well-oxygenated environments [29]. Growth factor signal transduction has also been suggested to enhance HIF-1α expression [30]. Genetic mutations that result in hyperactivation of oncogenic signal transduction pathways also enhance HIF-1α expression [31, 32].

The related functions of HIF-1α in fibrosis include stimulating excessive ECM, vascular remodeling and ineffective angiogenesis, further aggravating chronic hypoxia and deteriorating pathological fibrosis [33]. Chronic hypoxia in skeletal muscle pathology is an important feature of fibrosis. HIF-1α and TGF-β1 co-driven CCN2 overexpression contributes to the establishment of fibrosis [34]. It has also been reported that TGF-β1 induces HIF-1α stabilization [35]. In this study, we demonstrated that TGF-β1 enhanced HIF-1α expression in MYBPC3-KO fibroblasts, which could be reversed by SB-431542.

The switch of metabolism from oxidative phosphorylation to aerobic glycolysis (Warburg effect) and increased lactic acid production were observed not only in R495Q mutant pigs but also in MYBPC3-KO fibroblasts, which could be reversed by aerobic glycolysis inhibitor 2-DG. These observations are consistent with previous reports that aerobic glycolysis occurs under a wide range of fibrotic conditions [36]. For example, downregulation of fatty acid oxidation (FAO) and upregulation of glycolysis were found in fibrotic skin [37]. In addition, human keloid fibroblasts are characterized by higher rate of ATP biosynthesis, with glycolysis as their primary energy source, demonstrated by increased lactic acid production [38]. The Warburg switching in renal fibroblasts is the primary feature of fibroblast activation during renal fibrosis and that suppressing renal fibroblast aerobic glycolysis could significantly reduce renal fibrosis [39]. Previous studies also demonstrated that LPS promotes collagen synthesis in lung fibroblasts through aerobic glycolysis via the activation of PI3K-Akt-mTOR/PFKFB3 pathway [40]. Analysis of liver samples from patients with hepatocellular carcinoma (HCC) and patients with cirrhosis showed that the expression of glycolytic enzymes was up-regulated in precancerous cirrhotic livers and significantly associated with elevated risks for developing HCC [41]. These findings suggest that the metabolism switching is a fundamental feature of fibroblast activation in various disease conditions including HCM.

In conclusion, our studies suggest that *MYBPC3* deficiency in cardiac fibroblasts can promote their trans-differentiation into myofibroblasts via NF-κB/TGF-β1/HIF-1α/aerobic glycolysis signal cascade, contributing to premature myocardial fibrosis. These findings highlight the importance to consider cardiac fibroblasts as additional therapeutic target in future therapeutic development for HCM patients with *MYBPC3* mutations.

## Methods

### Ethics statement

All animal studies were approved by the Animal Welfare and Research Ethics Committee at Jilin University (SY201903015), and all procedures were conducted strictly in accordance with the Guide for the Care and Use of Laboratory Animals. All surgeries were performed under anesthesia, and every effort was made to minimize animal suffering.

### sgRNA vectors targeting mouse and pig MYBPC3 genes

The sgRNA oligonucleotides targeting mouse *Mybpc3* gene (Comate Bioscience, China) were annealed and cloned into pX330-U6-Chimeric_BB-CBh-hSpCas9 (Addgene #42230) and the sgRNA oligonucleotides targeting pig MYBPC3 gene were annealed and inserted into pBluescriptSKII+ U6-sgRNA(F + E) empty expression vector (Addgene #74707).

### Cell culture, transfection, and genotyping of cell clones

NIH3T3 (mouse embryonic fibroblast cells) and PFFs (porcine fetal fibroblasts) were cultured in DMEM (GIBCO) supplemented with 15% fetal bovine serum (FBS) at 37 °C and 5% CO_2_ in a humidified incubator. To generate R495Q mutant PFFs, approximately 3 × 10^6^ PFFs were electro-transfected with pCMV_AncBE4max plasmid (Addgene #112094) and MYBPC3-sgRNA plasmid (15 μg each) in 200 μL of Opti-MEM (GIBCO) using 2-mm gap cuvettes and a BTX ECM 2001 electroporator. The parameters for electrotransfection were as follows: 340 V, 1 ms, 3 pulses for 1 repeat. At 36 h after electrotransfection, the cells were plated into ten 10-cm dishes at a density of 4 × 10^3^ cells per dish. Single-cell colonies were picked and cultured in 48-well plates. When the plates reached 90% confluence, 50% of cells from each plate was lysed using 20 μL of lysis buffer (0.45% NP-40 plus 0.6% Proteinase K) for 60 min at 56 °C and 10 min at 95 °C to provide templates for genotyping with the following primers: pMYBPC3-JD-F, TCTTTGAGTCCATCGGCACC, and pMYBPC3-JD-R, CCCACAGTCAAGTCTGCGAT. The PCR conditions were 94 °C for 5 min; 94 °C for 30 s, 55 °C for 30 s, and 72 °C for 1 min for 35 cycles; 72 °C for 5 min; and hold at 16 °C. The MYBPC3-KO NIH3T3 cells were similarly generated except that the pX330-U6-Chimeric_BB-CBh-hSpCas9 with Mybpc3-sgRNA plasmid (30 μg) was used for electrotransfection. The following primers were used for genotyping of MYBPC3-KO NIH3T3 cells: mMYBPC3-JD-F, GAACAGGCAAACGAAGGACAG, and mMYBPC3-JD-R, TCTTGGTGCAGAAGAGGGGAA.

### Off-target assay

Potential off-target sites (OTSs) for each sgRNA were predicted by Cas-OFFinder (http://www.rgenome.net/cas-offinder/). OTS were analyzed via PCR and DNA sequencing to determine the target effects. The primer sequences used for analyzed the off-target activities are listed in Supplemental Tables S1 and S2.

### SCNT and euthanization

SCNT and embryo transfer were performed as described previously [42]. Briefly, recipient sows (100–120 kg, 9–10 months of age) were pre-anesthetized by subcutaneous or intramuscular injection of atropine (0.05 mg/kg). During operation, anesthesia was maintained by constant inhalation of 2% isoflurane. Subcutaneous injection of carprofen (2–4 mg/kg) was used for analgesia, once per day for three days starting from the day of operation.

Euthanization of the animals was carried by intravenous injection of a lethal dosage of pentobarbital sodium (100–200 mg/kg), followed by chest opening and tissue collection.

### Serum biochemical analysis of piglets

Serum samples were collected and measured by ELISA following the manufacturer’s instructions, in an infinite 2000 PRO Microplate Reader (Tecan, Switzerland). Samples were measured in triplicate, and the absorbance was monitored at 37 °C.

### Cell proliferation assay

Cell proliferation assay was detected by 5-ethynyl-2-deoxyuridine (EdU, RiboBio, China) following the manufacturer’s instructions. Briefly, cells were seeded into 96-well plates at a density of 1 × 10^5^ cells per well and incubated with 10 μM EdU for 1 h. After that, cells were fixed and stained with Hoechst 33342. Images were captured using the fluorescence microscope (Olympus BX51). EdU-positive cells were quantified via ImageJ (NIH, Bethesda, MD, USA) on unmanipulated TIFF images.

### Cell wound scratch assay

Cell wound scratch assay was performed as manufacturer’s instruction. Firstly, marker pen was used to draw a line at an interval of 1 cm behind the 6-well plate. Then, about 5 × 10^5^ cells were plated into 6-well plate. Then, a scratch wound was generated using a pipette tip and washed three times with PBS to remove the scratched cells. Subsequently, serum-free medium was added and cells were further cultured in a humidified incubator at 37 °C. Cells were stained with Calcein-AM (Dojindo, China) and imaged at 0, 6, 12, 24, and 48 h. Numbers of fluorescent cell corresponding to wound closure rate were calculated by Image-J software (NIH, Bethesda, MD, USA).

### Cell cycle analysis

Cell cycle was detected by *Cell Cycle and Apoptosis Analysis Kit* (Beyotime, China) following the manufacturer’s instructions. First, cells were washed twice with PBS and fixed in 70% cold ethanol overnight at 4 °C. And then stained with propidium iodide (PI) for 30 minutes in the dark at 37 °C. Finally, the DNA content was measured by fluorescence-activated cell sorting (FACS) instrument (BD Biosciences).

### Cell apoptotic analysis

Cell apoptosis was detected by Annexin V-FITC apoptosis detection kit (Beyotime, China). A total of 1 × 10^5^ cells were incubated with Annexin V-FITC and PI in the provided binding buffer for 30 min in the dark at 4 ˚C, and analyzed by fluorescence-activated cell sorting (FACS) instrument (BD Biosciences).

### Measurement of intracellular adenosine triphosphate (ATP)

Intracellular ATP level was detected by using Enhanced ATP Assay Kit (Beyotime, China). In accordance with the manufacturer’s instructions, cells were washed twice with ice-cold PBS, lysed using ice-cold ATP lysis buffer, and then centrifuged for 5 min to collect the supernatants. Then, ATP concentrations were measured by luminescence and ATP level was calculated according to standard curve with an infinite 2000 PRO Microplate Reader (Tecan, Switzerland). Samples were measured in triplicate, and the absorbance was monitored at 37 °C. All results were normalized to the total protein concentration.

### Measurement of glucose concentrations

Glucose concentrations were detected by *Tissue Cell Glucose Oxidase Assay Kit* (Pplygen, China) following the manufacturer’s instructions. The glucose concentrations were measured and calculated according to standard curve with an infinite 2000 PRO Microplate Reader (Tecan, Switzerland). Samples were measured in triplicate, and the absorbance was monitored at 37 °C. All results were normalized to the total protein concentration.

### Measurement of lactic acid concentrations

*Lactic acid concentrations were determined by* Lactic Acid assay kit (Nanjing Jiancheng Bioengineering Institute, China) according to the manufacturer’s instruction. The OD was measured by an infinite 2000 PRO Microplate Reader (Tecan, Switzerland). Samples were measured in triplicate, and the absorbance was monitored at 37 °C. All results were normalized to the total protein concentration.

### Cell viability assays (CCK8 assay)

Equal numbers of viable cells were plated in 96-well plates. Cells were incubated with 200 μL drug-supplemented medium, treated with DMSO (vehicle) at 0.1% or the following drug concentrations standardized to 0.1% DMSO final concentration. For NIH-3T3 cells, the treatment regimens were: 2-DG (MCE, China), 1 mM, 5 mM, 10 mM, 15 mM and 20 mM; SB-431542 (MCE, China), 5 μM, 10 μM, 50 μM,100 μM and 200 μM; Oltipraz (MCE, China), 5 μM, 10 μM, 50 μM,100 μM and 200 μM; PDTC (MCE, China), 5 μM, 10 μM, 50 μM,100 μM and 200 μM; After incubation for 24 h, 48 h or 72 h, cell viability was measured using a Cell Counting Kit-8 (CCK8) assay (Dojindo, China) according to the manufacturer’s instructions. The optical density at 450 nm (OD450 nm) was measured using an infinite 2000 PRO Microplate Reader (Tecan, Switzerland). Samples were measured in triplicate, and the absorbance was monitored at 37 °C (Supplemental Fig. S6).

### Measurement of TGF-β1, CCL2, IL-1β, TNF-α, and IL-6

Cell culture supernatant and cell lysates were measured by ELISA kits (Boster, China) following the manufacturer’s instructions. The OD was measured by an infinite 2000 PRO Microplate Reader (Tecan, Switzerland). Samples were measured in triplicate, and the absorbance was monitored at 37 °C.

### Quantitative reverse transcription PCR (RT-PCR)

For detection of relative mRNA expression of genes, total RNA was isolated by TRNzol-A^+^ Reagent (TIANGEN, China) following the manufacturer’s recommendations. One μg RNA was reverse-transcribed (RT) to generate cDNA using a FastKing RT Kit (with gDNase) (TIANGEN, China) according to the manufacturer’s manual. The fluorescence intensity and amplification plots were analyzed by a BIO-RAD iCycler Thermal Cycler with iQ5 Optical Module for RT-PCR (Bio-Rad, ABI 7500, iQ5). The primers used in RT-PCR are listed in Supplemental Table S3.

### Western blotting (WB)

Western blotting was performed as described manufacturer’s instructions. Equal amounts of 40 μg proteins were separated through SDS-PAGE on a 10% separating gel, and the protein bands were electrophoretically transferred to polyvinylidene fluoride (PVDF) membranes. The protein bands were detected with the ECL-Plus Western blotting reagent. The primary and secondary antibodies involved in the process were shown in Supplemental Table S4.

### H&E staining, Masson trichrome staining, and fibrosis quantification analysis

Fresh heart muscle tissues were fixed in 4% PFA, embedded in paraffin, and sectioned at 5 μm. HE staining and Masson staining were performed with standard techniques. For fibrosis analysis, nine non-overlapping pictures (400X) were randomly taken from each section and then calculated fibrotic area (blue) and total tissue area by ImageJ software (NIH, Bethesda, MD, USA).

### Statistical analysis

All data are expressed as the means ± standard error of the mean (SEM). Statistical differences were determined by unpaired Student’s t-test for two group comparisons, and one-way ANOVA with Bonferroni’s post-tests for multiple group comparisons. All statistical analyses were completed using GraphPad Prism 7.0 software.

## Supplementary information

SUPPLEMENTAL MATERIAL

## Data Availability

All datasets generated and analyzed in this study are included in this published article and its Supplementary Information files. Additional data are available from the corresponding author on reasonable request.
