# Supplementary material for: MYBPC3 deficiency in cardiac fibroblasts drives their activation and contributes to fibrosis
Source: Cell Death Dis. 2022 Nov 10;13(11):948. doi: 10.1038/s41419-022-05403-6 (PMC9649783; doi:10.1038/s41419-022-05403-6)
Supplement: Supplementary file 1 — SUPPLEMENTAL MATERIAL [file 41419_2022_5403_MOESM1_ESM.pdf]

Supplemental Information

Supplemental Table S1： List of putative off-target sites homologous to sgRNA.

PAM sequences are labeled in blue. Base substitutions are shown in red.

**Mouse-mybpc3-sgRNA**	GGAGCCTGCAATGACCGCGT AGG
**OTS1**	GGAGCCTGGAATGACAGCTT TGG
**OTS2**	AGAGCCTGCACTGGCCGCGT TGG
**OTS3**	GGAGCCTGTAATGACCCAGT GGG
**OTS4**	GGAGCCTGCTATGTCCCCGT GGG
**OTS5**	TGAGCCTGCAATGATCACGT GGG
**Pig-*mybpc3*-sgRNA**	CTCCCGGGTCAGCTCCACCC CGT
**OTS1**	ATCCCAGCACAGCTCCACCC TGG
**OTS2**	CTCCCGAGCTAGCACCACCC GGG
**OTS3**	CCCACTGGCCAGCTCCACCC TGG
**OTS4**	CATTCGGGACAGCTCCACCC TGG
**OTS5**	TTCCCGCAGCAGCTCCACCC CGG
**OTS6**	CCCGCAGGCCAGCTCCACCC TGG

Supplemental Table S2：List of primers for PCR amplification of off-target sites.

Primers	Sequences (5' to 3')	Amplicon (bp)
**Mouse-MYBPC3- sgRNA-OTS1**	AACGTGCTCATGCCTCTCAG	624
	GCAGACAGAATGCACACTGG	
**Mouse-MYBPC3- sgRNA-OTS2**	AATCCCAGAACGAAGGCCTG	783
	GCAAAGAGGACACTGAGGCT	
**Mouse-MYBPC3 - sgRNA-OTS3**	CTCAACCTGTGGGTCGAGAC	686
	CAGCAGGACCCATGGAAAGT	
**Mouse-MYBPC3 - sgRNA-OTS4**	GTTGGCTCCCTTGCTTCTCT	754
	CATGGAGAGATCCGAGCCAC	
**Mouse-MYBPC3 - sgRNA-OTS5**	CCCTGTTCACGGCATCTCAT	713
	CCGCCGCCATCAGTATAACT	
**Pig-MYBPC3- sgRNA-OTS1**	GTCCCAGGGAAGAAACCAGG	863
	TTGTCCAGTGTGGCTTGGTT	
**Pig-MYBPC3- sgRNA-OTS2**	GGTTACAGACACGGCTTGGA	936
	GGAGGCACCAAGGAAGTTGA	
**Pig-MYBPC3- sgRNA-OTS3**	CAGAGATCCTCCATCACCGC	811
	AGCTCTGAGTCACCTCCCTT	
**Pig-MYBPC3- sgRNA-OTS4**	GCCCTCTCTCATAGCCACAC	698
	GGTCGTGGAAACAGGCTACA	
**Pig-MYBPC3- sgRNA-OTS5**	AGTGAGGATGCAGCGTTTGA	923
	AACCTATCCCTGCCCTTTGC	

**Pig-MYBPC3- sgRNA-OTS6**	CTGGCCTCTCTGAGCATCTG	840
	TCCACACAGCTCTGGAAACC	

Supplemental Table S3：List of the primers used in RT-PCR.

Primers	Sequences (5' to 3')	Amplicon (bp)
**Mouse-MYBPC3-RT**	GAACACTACCGACGCACTCA	176
	AGGGCCTTGTATTTGGGTGG	
**Mouse-HIF-1α-RT**	CCAGCCTAACAGTCCCAGTG	240
	GGAGGGCTTGGAGAATTGCT	
**Mouse-TGF-β1-RT**	ACTGGAGTTGTACGGCAGTG	123
	GGGGCTGATCCCGTTGATTT	
**Mouse-GCK-RT**	AGGTAGAGCAGATCCTGGCA	143
	CACGTAGGTGGATGAGAGCTC	
**Mouse-PFKM-RT**	TTGGAGCTGATGGAAGGCAG	297
	ATCGCGAATGGTGAAGGGTT	
**Mouse-LDHA-RT**	GGAGCAGTGGAAGGAGGTTC	293
	TGTCTGCGCTCTTCTTCAGG	
**Mouse-COL1A1-RT**	CCCAGTGGCGGTTATGACTT	117
	CTCAAGGTCACGGTCACGAA	
**Mouse-α-SMA-RT**	CCCAATGGTGAGACGTGGAA	198
	CTTGGGTCCCTCGACTCCTA	
**Mouse-ACTB-RT**	TCGAGACCTTCAACACCCCA	101
	GTCTCCGGAGTCCATCACG	
**Pig-MYBPC3-RT**	CTGTGGTGCCAATCGCTCTT	177
	GGACGTCCAGACGTAGCTTG	

**Pig-HIF-1α-RT**	TACTCATCCGTGCGACCATG	257
	CCAAGCACGTCATAGGTGGT	
**Pig-TGF-β1-RT**	GGCCAGATTCTGTCCAAGCT	209
	ATTTGGTTGCCGCTTTCCAC	
**Pig-GCK-RT**	CCGACTTCCTGGACAAGCAT	181
	CGTTTGATGGCATCTCGCAG	
**Pig-PFKM-RT**	AGGGCCTTGTGATCATTGGG	202
	TGCTTAATGCGGTCACAGGT	
**Pig-LDHA-RT**	GTCATGGGTGGATCCTTGGG	141
	TGTGAACCGCTTTCCAGTGT	
**Pig-COL1A1-RT**	CCTGGACGCCATCAAAGTCT	125
	AGACGTGCCTCTTGTCCTTG	
**Pig-α-SMA-RT**	AAGATCAGGACAGGCTTGCC	226
	GCATTAGCTGGGGTGACTGT	
**Pig-GAPDH-RT**	ATCCTGGGCTACACTGAGGA	130
	TGTCGTACCAGGAAATGAGCT	

Supplemental Table S4：List of the primary and secondary antibodies used in this study.

Antibodies	Host	Dilutions	Supplier
Anti-MYBPC3 Antibody (E-7)	Mouse	1:1000	Santa Cruz Biotechnology
MYL (F-5)	Mouse	1:1000	Santa Cruz Biotechnology
Anti-VIM Antibody	Mouse	1:1000	BOSTER
Rabbit Anti-Troponin T/TNNT2	Rabbit	1:1000	BOSTER
Anti-TGF beta 1 Antibody	Rabbit	1:1000	BOSTER
Anti-COL1A1 Antibody	Rabbit	1:1000	BOSTER
a-SMA Antibody (monoclonal,1A4)	Mouse	1:1000	BOSTER
Anti-GCK Antibody	Rabbit	1:1000	BOSTER
PFKM Polyclonal antibody	Rabbit	1:1000	Proteintech
Anti-LDHA Antibody	Rabbit	1:1000	BOSTER
Anti-HIF1A Antibody	Rabbit	1:1000	BOSTER
Phospho-NF-κB p65 (Ser536) (93H1)	Rabbit	1:1000	Cell Signaling Technology
NF-κB p65 (D14E12)	Rabbit	1:1000	Cell Signaling Technology
β-Actin Mouse Monoclonal Antibody	Mouse	1:1000	Beyotime
HRP-labeled Goat Anti-Mouse IgG(H+L)	Goat	1:1000	Beyotime
HRP-labeled Goat Anti-Rabbit IgG(H+L)	Goat	1:1000	Beyotime
Alexa Fluor 488 Goat anti-Mouse -IgG(L+H)	Goat	1:500	Proteintech

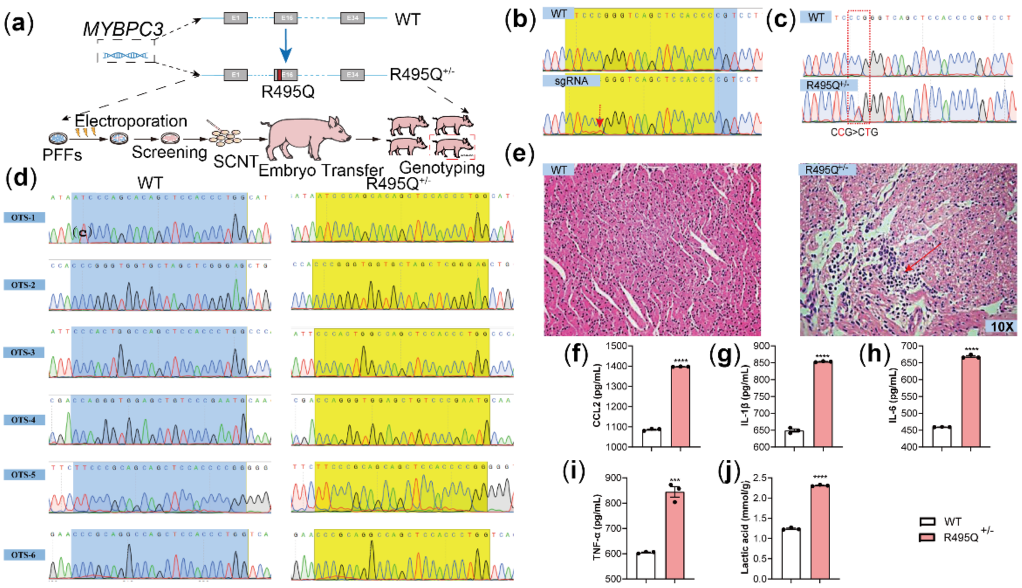

Supplemental Figure S1. Sequence analysis of pig *MYBPC3* gene and design of CBEs-targeting strategy. (a) The flowchart to generate MYBPC3-R495Q mutant pigs. (b) Sanger sequencing traces of PCR amplicons from WT and electrotransfected PFFs. The target base is labeled with a red arrow. (c) Gene targeting analysis by T-cloning and Sanger sequencing in all piglets. (d) Analysis of off-target activities at the top predicted off-target sites. (e) The H&E-stained images of WT and R495Q$^{+/-}$ pig heart sections. (f-i) Serum levels of CCL2, IL-1β, IL-6 and TNF-α in WT and R495Q$^{+/-}$ pigs (n=3). ***P<0.001, ****P<0.0001. (j) Lactic acid measurements in heart tissues of WT and R495Q$^{+/-}$ pigs (n=3). ****P<0.0001.

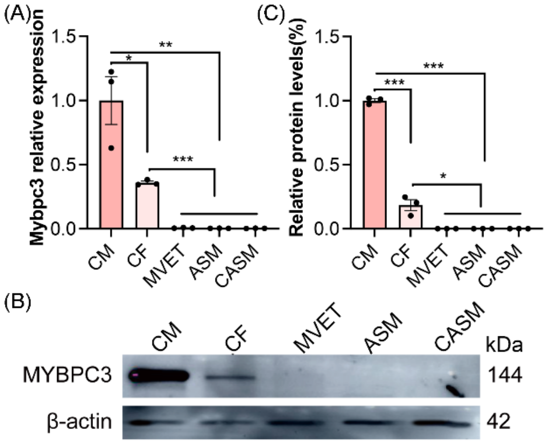

Supplemental Figure S2. Expression of MYBPC3 in major cell types in mouse heart. (A) The relative expression of *Mybpc3* transcript in mouse cardiac myocytes (CM), fibroblasts (CF), microvascular endothelial cells (MVET), aortic smooth muscle cells (ASM) and coronary artery smooth muscle cells (CASM). *P<0.05, **P<0.01, ***P<0.001, n=3. (B, C) Western blot and densitometry analysis of MYBPC3 expression in mouse heart cells.

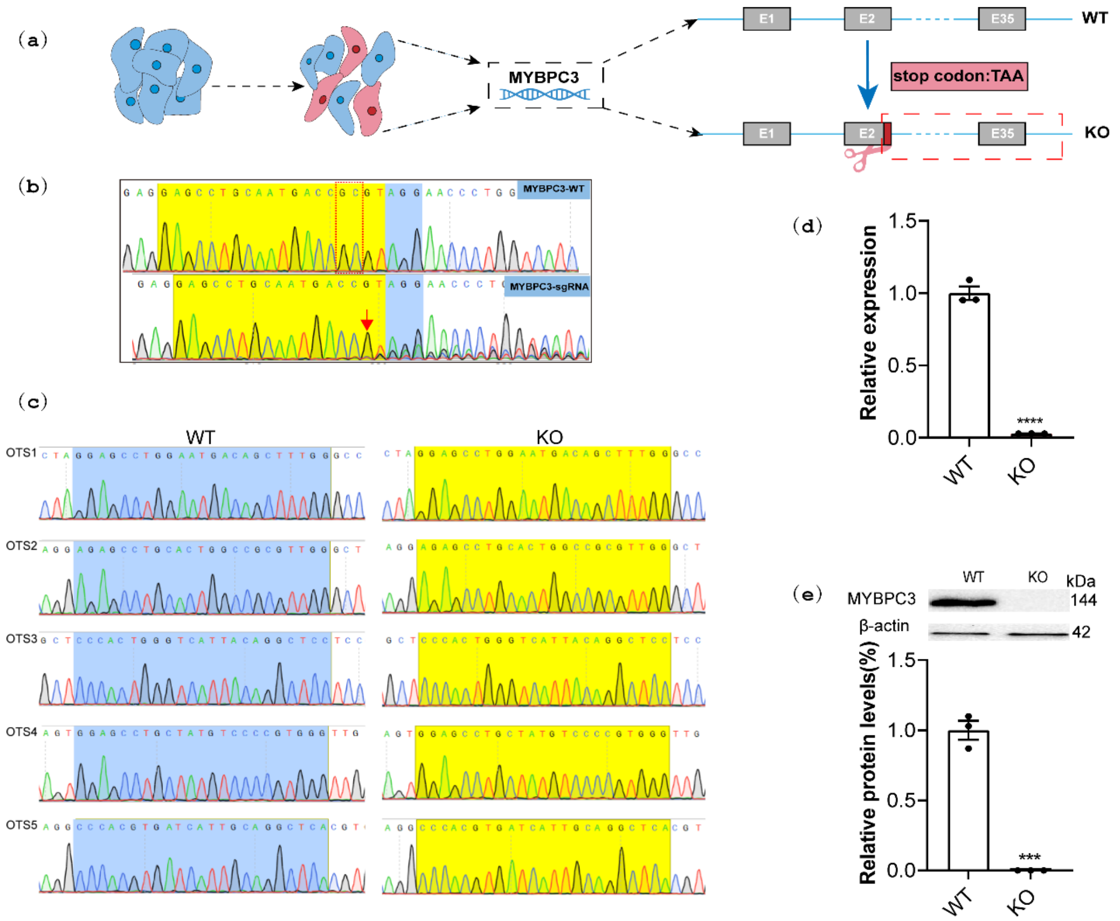

Supplemental Figure S3. The design of CRISPR-targeting strategy in fibroblasts.

(a) Schematic showing the targeting strategy to generate MYBPC3-KO NIH3T3 fibroblasts. (b) Sanger sequencing traces showing the CRISPR/Cas9-engineered indels in mouse *Mybpc3* gene. PAM is highlighted in blue. (c) The off-target activity analysis. (d, e) Quantitative RT-PCR and Western blot analysis of *Mybpc3* expression in WT and MYBPC3-KO NIH3T3 cells (n=3). ***P<0.001, ****P<0.0001.

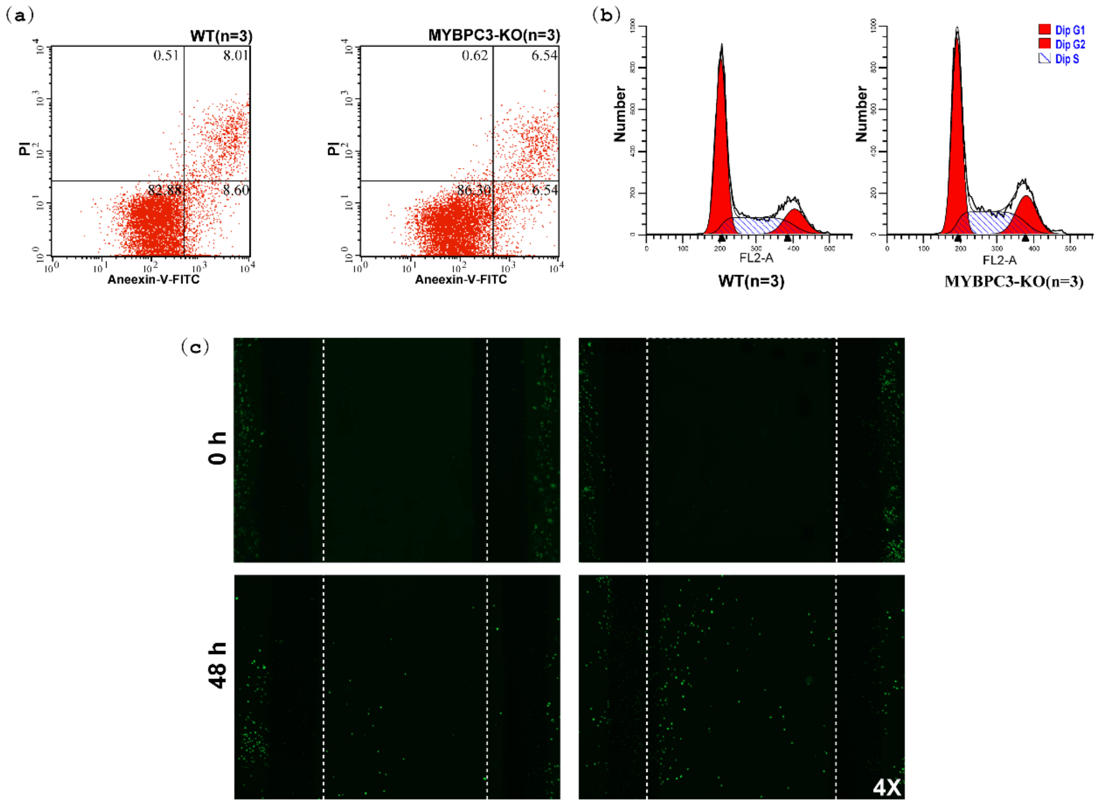

Supplemental Figure S4. The impact of MYBPC3 disruption on apoptosis, cell cycle and migration of NIH3T3 fibroblasts. (a-b) Analysis of apoptosis (a) and cell cycle (b) in WT and MYBPC3-KO NIH3T3 fibroblasts by fluorescence-activated cell sorting (FACS). (c) Cell migration analysis in WT and MYBPC3-KO fibroblasts by the wound scratch assay.

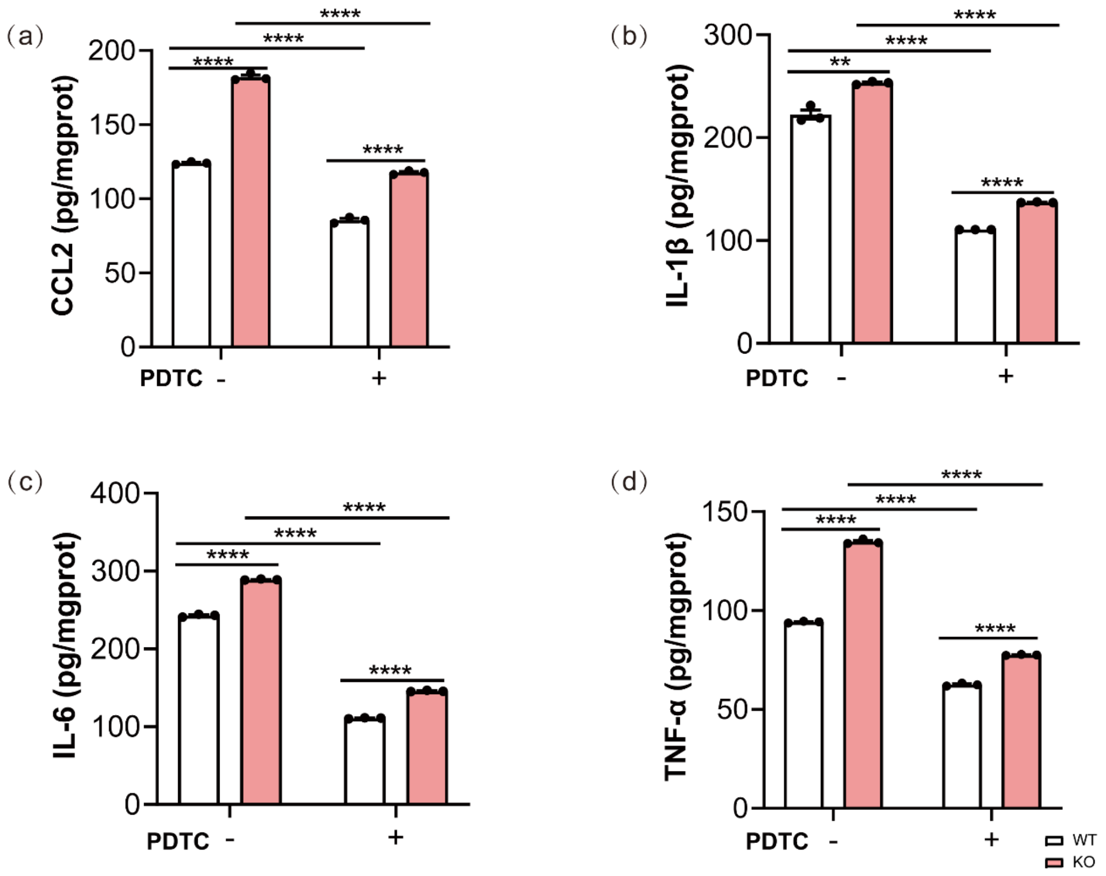

Supplemental Figure S5. Activated NF-κB signaling pathway in MYBPC3-KO fibroblasts. Measurements of CCL2, IL-1β, IL-6 and TNF-α in cell lysates of WT and MYBPC3-KO NIH3T3 fibroblasts with or without PDTC treatment (n=3). **P<0.01, ****P<0.0001.

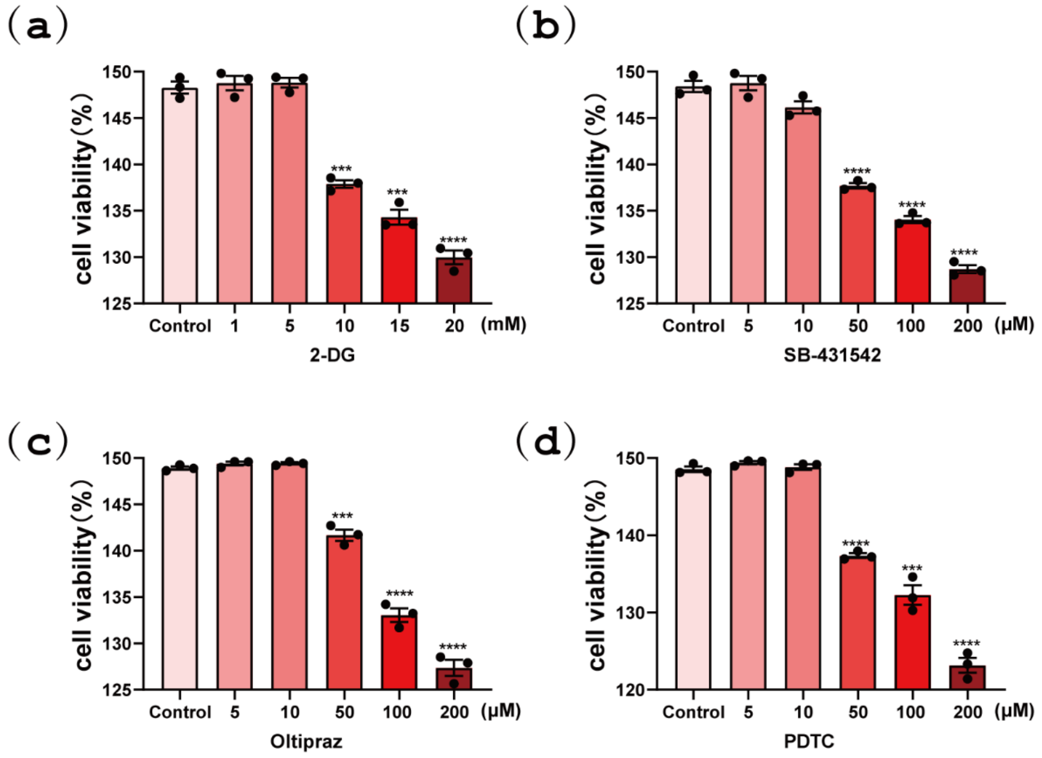

Supplemental Figure S6. Concentration-dependent cell toxicity of 2-DG, SB-431542, Oltipraz and PDTC. The cell toxicity of 2-DG, SB-431542, Oltipraz and PDTC was analyzed by CCK8 assays (n=3). ***P<0.001, ****P<0.0001.

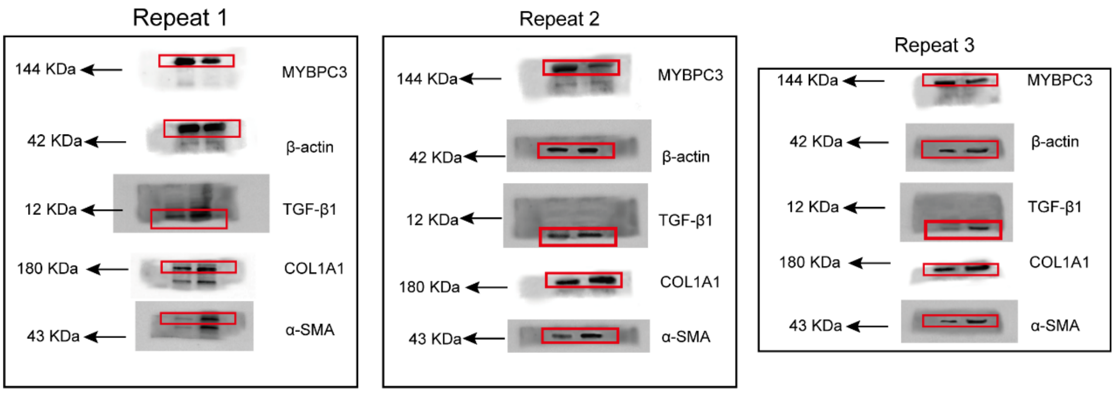

Supplemental Figure S7 related to Figure 1B. Uncropped immunoblots of MYBPC3, TGF-β1, COL1A1 and α-SMA with β-actin obtained from heart tissue lysate. All experiments were performed three times.

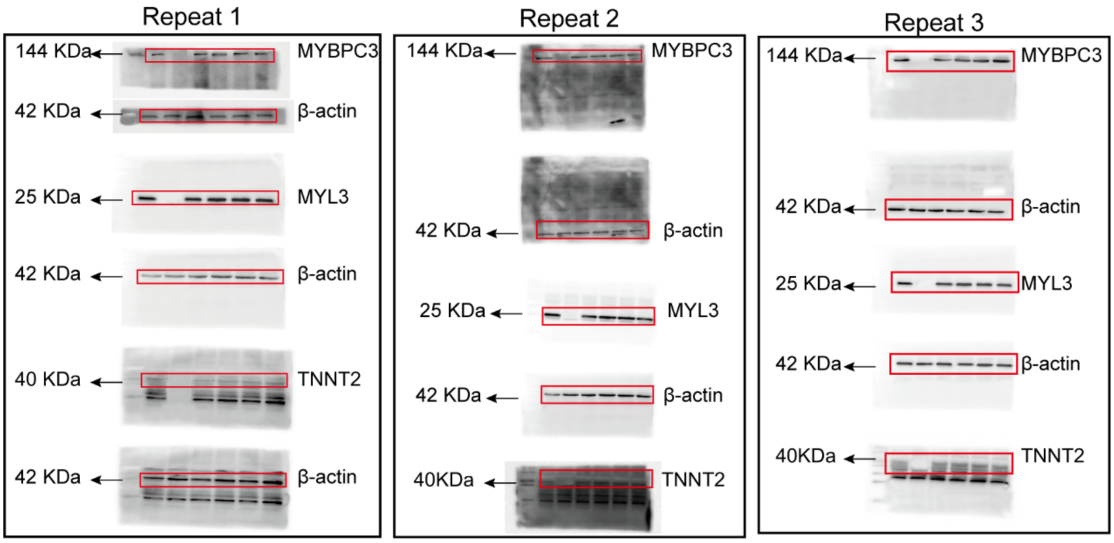

Supplemental Figure S8 related to Figure 2B. Uncropped immunoblots of MYBPC3, MYL3, TNNT2 with β-actin obtained from fibroblasts cell lysate. All experiments were performed three times.

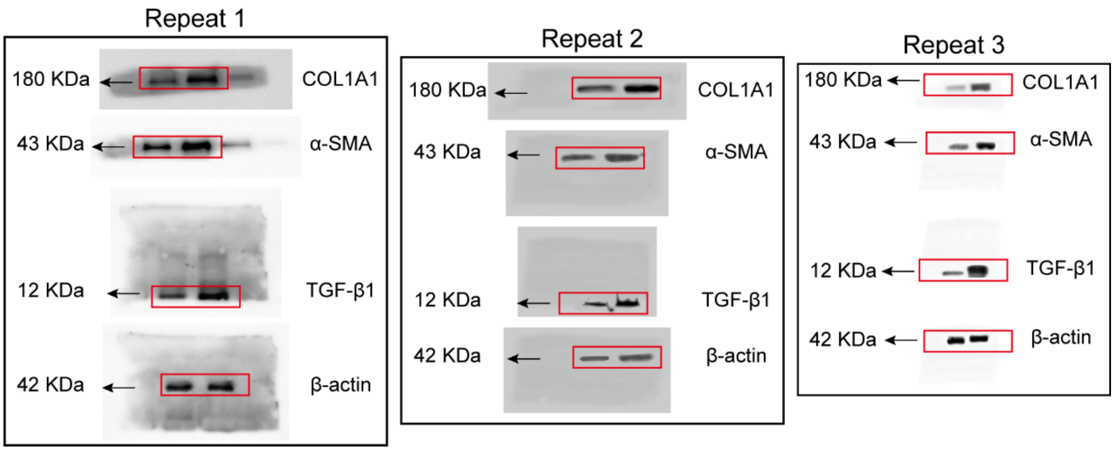

Supplemental Figure S9 related to Figure 3B. Uncropped immunoblots of COL1A1, α-SMA and TGF-β1 with β-actin obtained from NIH-3T3 cell lysate. All experiments were performed three times.

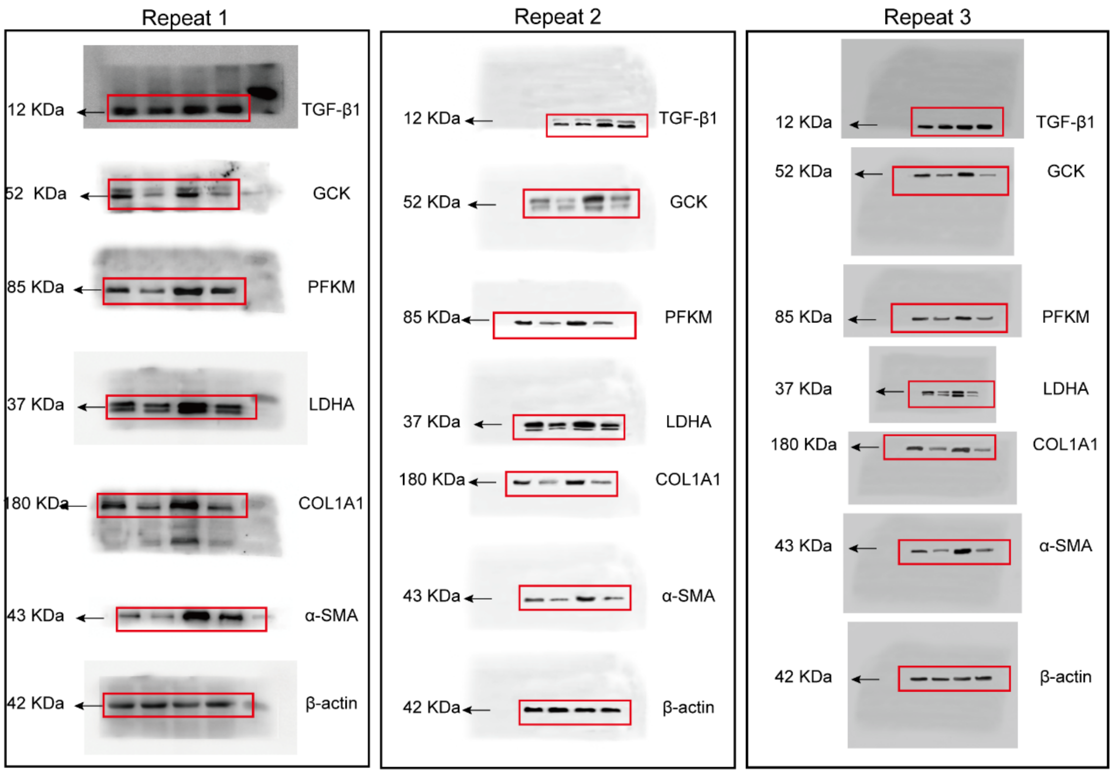

Supplemental Figure S10 related to Figure 4G. Uncropped immunoblots of TGF-β1, GCK, PFKM, LDHA, COL1A1, α-SMA and with β-actin obtained from NIH-3T3 cell lysate. All experiments were performed three times.

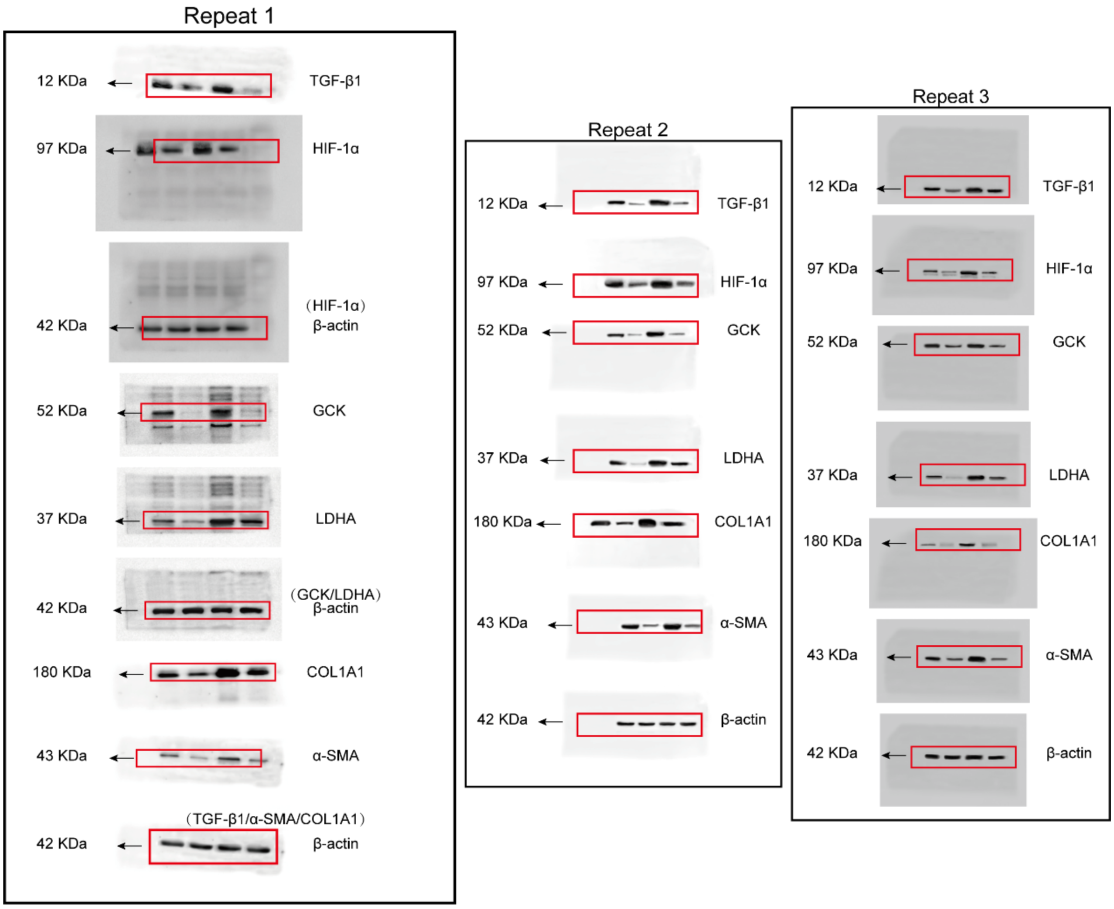

Supplemental Figure S11 related to Figure 5A. Uncropped immunoblots of TGF-β1, HIF-1α, GCK, LDHA, COL1A1, α-SMA and with β-actin obtained from NIH-3T3 cell lysate. All experiments were performed three times.

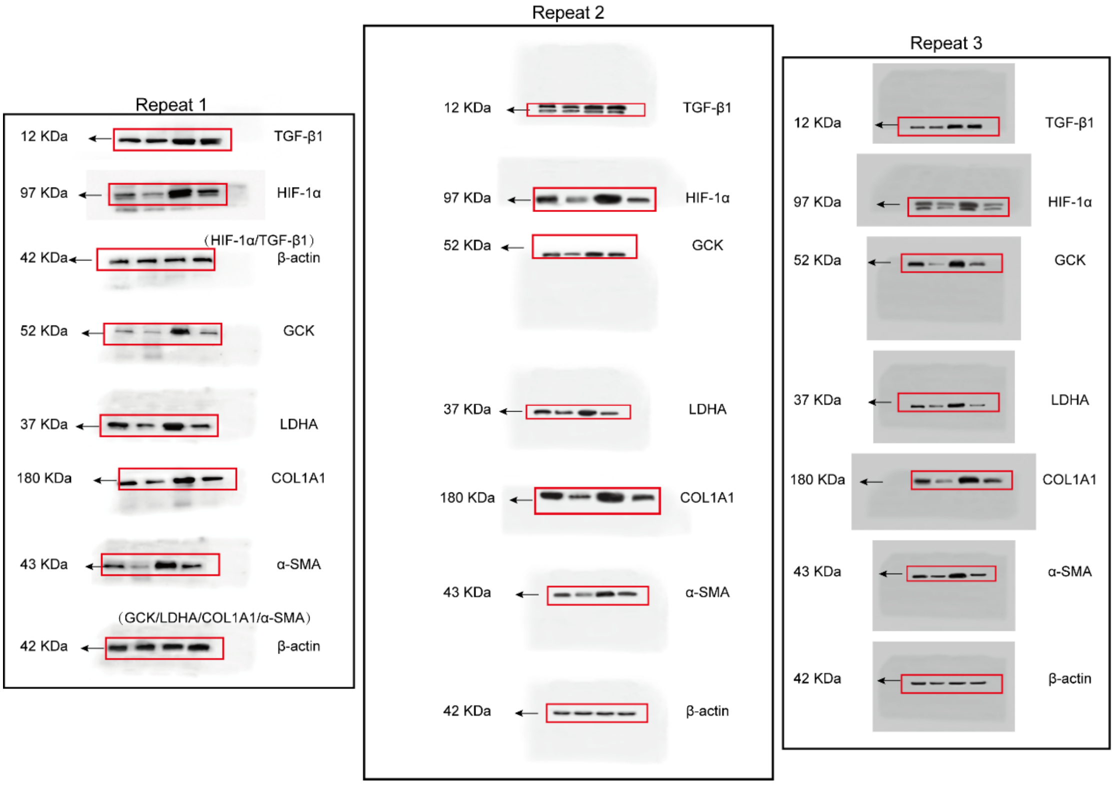

Supplemental Figure S12 related to Figure 5B. Uncropped immunoblots of TGF-β1, HIF-1α, GCK, LDHA, COL1A1, α-SMA and with β-actin obtained from NIH-3T3 cell lysate. All experiments were performed three times.

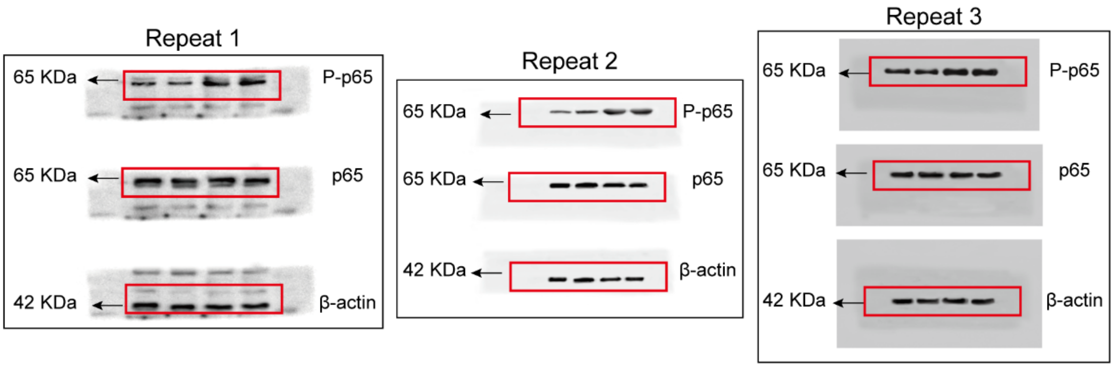

Supplemental Figure S13 related to Figure 6A. Uncropped immunoblots of P-p65 and p65 with β-actin obtained from NIH-3T3 cell lysate. All experiments were performed three times.

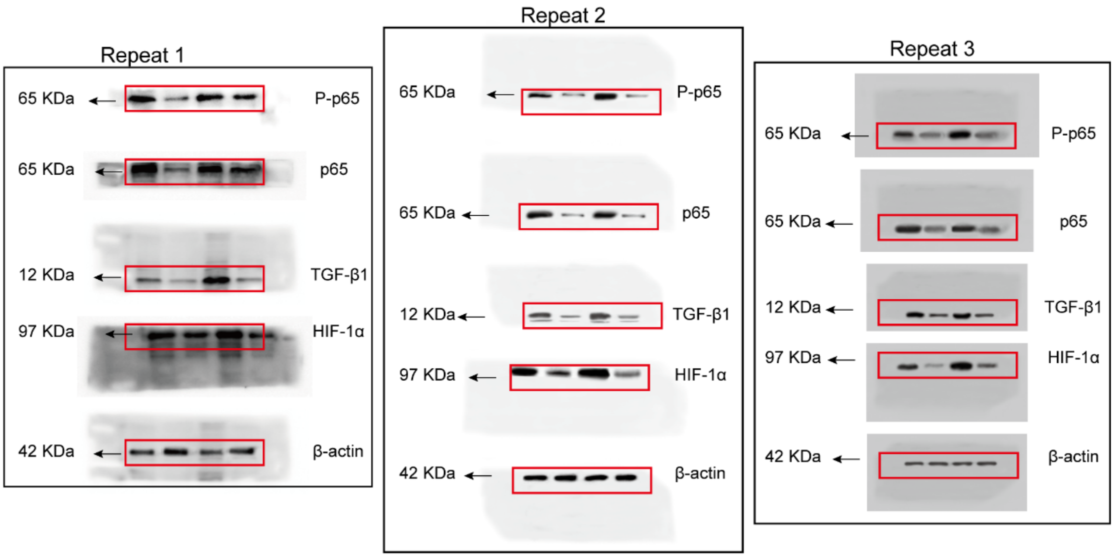

Supplemental Figure S14 related to Figure 6G. Uncropped immunoblots of P-p65, p65, TGF-β1 and HIF-1α with β-actin obtained from NIH-3T3 cell lysate. All experiments were performed three times.

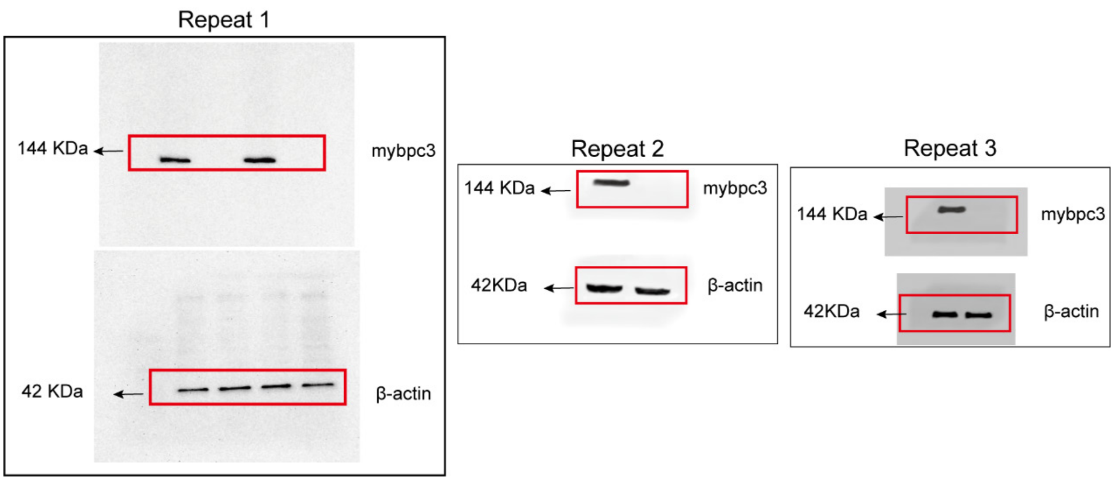

Supplemental Figure S15 related to Supplemental Figure S3e. Uncropped immunoblots of MYBPC3 with β-actin obtained from NIH-3T3 cell lysate. All experiments were performed three times.
